# Left Atrial Appendage Thrombus in Low CHA2DS2VASc Score in Persistent Atrial Fibrillation

**DOI:** 10.7759/cureus.81160

**Published:** 2025-03-25

**Authors:** Ezelea Elwina Walter Sandosam, Mohd Khairi Othman, Krishinan Saravanan, Zurkurnai Yusof, W Yus Haniff W Isa

**Affiliations:** 1 Internal Medicine, Universiti Sains Malaysia School of Medical Sciences, Kota Bharu, MYS; 2 Cardiology, Hospital Sultanah Bahiyah, Alor Setar, MYS; 3 Cardiology, Universiti Sains Malaysia School of Medical Sciences, Kota Bharu, MYS

**Keywords:** anticoagulant, left atrial appendage thrombus, persistent atrial fibrillation, pulmonary vein isolation, stroke

## Abstract

Atrial fibrillation is a commonly encountered arrhythmia, and its prevalence has increased over the years. Atrial fibrillation is the major cause of cardioembolic stroke, which is a debilitating complication of this arrhythmia. The most common site for this embolus is the left atrial appendage (LAA). CHA_2_DS_2_VASc score is the current scoring system that is used to stratify patients with atrial fibrillation on the risk of developing a stroke. Oral anticoagulants, either vitamin K antagonists or direct oral anticoagulants, are the treatment options in patients with a CHA_2_DS_2_VASc score of more than 2. Ironically, despite LAA being the most common site for thrombus formation, it is not included in this scoring system. Hence, patients with low CHA_2_DS_2_VASc scores still have a high risk of stroke with the presence of LAA thrombus alone. Herein, we report a case of a 51-year-old male individual who was diagnosed with atrial fibrillation-mediated cardiomyopathy complicated with LAA thrombus. An oral anticoagulant was not started during the initial diagnosis as his stroke risk was low based on the CHA_2_DS_2_VASc score. His score was 1. Incidentally, a huge LAA thrombus was detected before AF cardioversion for the rhythm control approach. Subsequently, he was managed using a direct oral anticoagulant and rhythm control approach. This case illustrates patients with low CHA_2_DS_2_VASc scores still can develop left atrial appendage thrombus, which could lead to a cardioembolic stroke. A careful assessment of stroke risk, including LAA thrombus risk assessment in patients with low CHA_2_DS_2_VASc scores is needed, to reduce the risk of thromboembolic stroke.

## Introduction

Atrial fibrillation (AF) is an arrhythmia that is commonly encountered, and the prevalence of AF varies depending on age. It was reported that the prevalence of atrial fibrillation is 0.5% among patients aged < 40 years old, 5% among patients aged >65 years, and 10% among the geriatric population [1]. AF is commonly associated with thromboembolic stroke, and CHA_2_DS_2_VASc score is used to risk stratify patients for stroke prevention using oral anticoagulants, either vitamin K antagonist (VKA) or direct oral anticoagulant (DOAC) [2]. The most common site for this embolus is the left atrial appendage, which has been seen in up to 90% of strokes caused by atrial fibrillation, which is not included in the CHA_2_DS_2_VASc score [3]. In AF, LAA thrombus formation is involved in complex pathophysiology, which predisposes to thrombus formation, and with a low CHA_2_DS_2_VASc score, stroke risk is still high. Herein, we report a case of a patient with a left atrial appendage thrombus despite having a low CHA_2_DS_2_VASc score and a review of the pathophysiology of the formation of LAA thrombus.

## Case presentation

We report a case of a 51-year-old male individual with a history of thyroidectomy done in 2022 for goiter. He was previously well without any symptoms until he started to have intermittent palpitations for a period of three weeks, which was associated with paroxysmal nocturnal dyspnea and orthopnea. This symptom was progressively worsening for three weeks and limited his daily activities. Otherwise, there were no other significant symptoms. There were no neurology-associated symptoms. Upon examination, blood pressure (BP) was 115/85 mmHg, and pulse rate (PR) was 110-120 bpm, which was irregular. The jugular venous pressure was elevated with fine crepitations upon lung auscultation and pitting pedal edema in both lower limbs. Upon neurological examination, higher mental function was intact, and no focal neurological deficit was noted.

**Figure 1:**
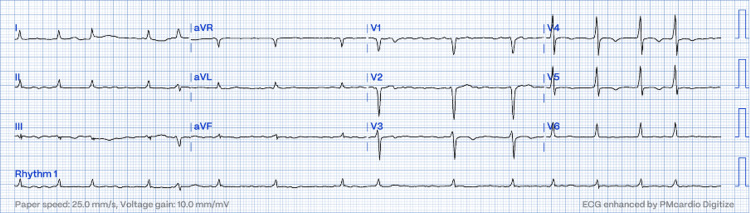
ECG showed absence P wave with irregularly irregular R-R interval.

His ECG showed atrial fibrillation with a rate of 110-120 bpm (Figure 1).

His blood investigation parameters, including thyroid function test, were within normal range. Transthoracic echocardiography showed a left atrium (LA) of 4.64 cm based on an anteroposterior diameter, dilated left ventricle with left ventricular ejection fraction (LVEF) of 30%. His diastology assessment showed grade 2 diastolic dysfunction. Subsequently, the initial diagnosis was de novo heart failure with atrial fibrillation. He was managed based on the four pillars of guideline-directed medical therapy (GDMT) for heart failure. Coronary angiography was performed as part of the diagnosis workup for heart failure etiology, which showed mild coronary artery stenosis. Hence, an atrial fibrillation-related cardiomyopathy diagnosis was made.

**Figure 2:**
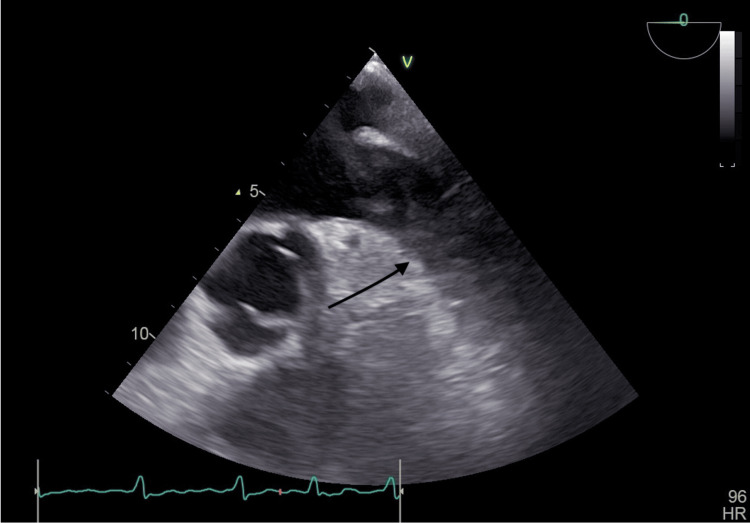
TOE midesophageal 0 degree showed presence of mobile and huge LAA clot (shown by black arrow) about one third of LAA. There was presence of spontaneous echo contrast and freely mobile LAA thrombus. The LAA opening orifies diameter was dilated measured 3.4 cm. TOE, transesophageal echocardiography; LAA, left atrial appendage.

The initial strategy of this patient was rhythm control using direct cardioversion (DCCV) given acceptable LA size. In preparation for cardioversion, transesophageal echocardiography (TOE) was performed, and the presence of a dilated left atrial appendage with a huge, freely mobile LAA thrombus and spontaneous echo contrast was noted (Figure 2). Hence, a decision to abandon the DCCV was made, and T. Apixaban 5 mg BD was initiated. A brain MRI was performed due to the presence of LAA thrombus, which showed bilateral small vessel disease and old infarct changes at the basal ganglia.

**Figure 3:**
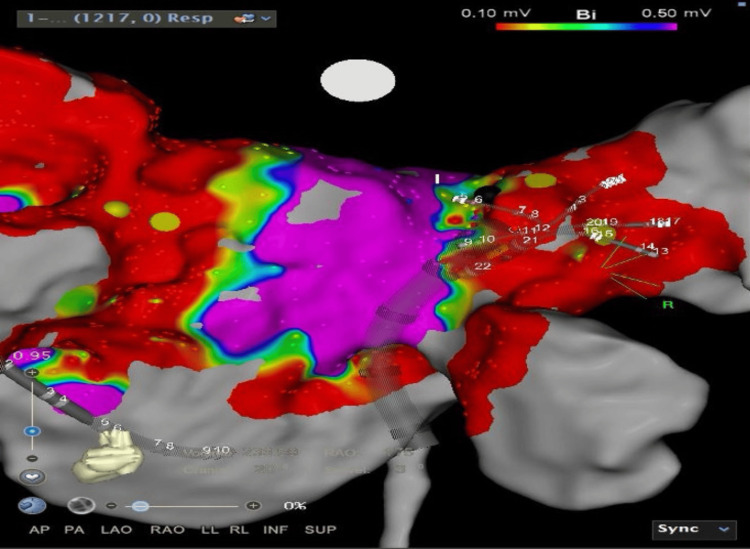
Electroanatomical figure showed all pulmonary veins was isolated post radiofrequency ablation. The red region signify low voltage area (presence of scar) and blue region signify high voltage area (healthy tissue). The was presence of line of block at the all pulmonary veins antrum shown by combination red and blue color.

Six weeks later, he was scheduled for pulmonary vein isolation of AF ablation, and pre-procedure TOE showed a resolution of LAA thrombus. He underwent wide antral circumferential ablation (WACA) using point-by-point radiofrequency ablation (Figure 3) and reverted to sinus rhythm. During serial follow-ups, he maintained a sinus rhythm and was self-monitored using a smartwatch. Serial transthoracic echocardiography showed his LVEF improved after four months post-procedure, and his functional class was New York Heart Association (NYHA) 1.

## Discussion

The risk of stroke is estimated by the CHA_2_DS_2_VASc score, which consists of congestive heart failure, hypertension, age, diabetes, history of stroke, vascular disease, and sex category in AF [4]. Current guidelines recommend oral anticoagulation for AF patients with a CHA_2_DS_2_VASc score of 2 or more [2]. In patients with low thrombosis based on CHA_2_DS_2_VASc score, it was reported the annual stroke was about 3% [4]. 

In AF, abnormal blood flow in the left atrium develops due to disorganized electrical signals, absence of coordinated atrial contraction, endothelial dysfunction, and other prothrombotic conditions that lead to thrombus formation [3]. The LAA is the most prominent site of LA thrombus, contributing 90% of thrombus in this location [5]. It has been known that three characteristics of LAA are associated with LAA thrombus and ischemic stroke: shape, size, and presence of fibrosis [3].

Using morphological characteristics, LAA was divided into four classical types: chicken wing, cactus, windsock, and cauliflower. Different LAA characteristics contribute to different risks of developing stroke, higher in cauliflower (18%) and lower in chicken wing (4%) morphology based on CT and MRI imaging [3]. Another study proves that cauliflower LAA morphology is associated with a higher risk of silent cerebral ischemia. In a patient with a low to intermediate risk of stroke, chicken wing LAA morphology is associated with a lower risk than non-chicken wing morphology [6]. The number of LAA lobes is an independent risk factor with moderate predictive value for LA thrombus formation [7].

Besides the morphology and lobe, the LAA size also plays an important role in predicting stroke. A multiple imaging modalities study was conducted to assess the LAA size and predict stroke [3]. It showed that higher LAA volume, larger LAA depth, larger LAA orifice, and extensive trabeculation are associated with a higher risk of stroke [3].

LAA fibrosis also plays an integral part in the development of LAA thrombus. LAA fibrosis is associated with reduced LAA flow velocities, which propagate to impaired blood flow in LAA, leading to thrombus development [8].

Apart from the LAA morphology, LAA function assessment based on transesophageal echocardiography has been associated with a higher risk of stroke. It has been shown from the Stroke Prevention in Atrial Fibrillation (SPAF)-III trial, that peak LAA anterograde flow velocity <20 cm/s is associated with thrombus [9]. Furthermore, among patients with LAA flow velocity < 11 cm/s the risk is higher [3,6]. A study done by Lee et al. showed that increased LAA orifices with decreased LAA emptying velocity have a significant risk of stroke [6]. A multimodal imaging assessment done by Beinart et al. showed an LAA volume of 22.9±9.6 cm^3^ and a larger LAA volume of 3.76±0.9 cm^3 ^[10]. In a similar study, LAA neck measurement using the short and long axes showed the strongest correlation with stroke risk [3]. Another study done by Burrell et al. assessing the LAA volume using MRI showed that LAA volume > 34 cm^3 ^ is associated with a higher risk of stroke [11]. It was found that LAA opening area and end-diastolic volume are associated with an increased risk of stroke [11]. 

It was reported that an opening area of more than 3.5 cm^2^ is associated with higher risk. LAA end-systolic volume based on transesophageal echocardiography with more than 18.54 ml and LAA end-diastolic volume of more than 9.49 ml is associated with a higher risk of thrombosis formation [12]. Left atrial diameter using transthoracic echocardiography has been studied to assess the risk of LAA clot, and it was found that a diameter of 4.35 cm has a better predictive value for LAA clot (sensitivity 84.4%, specificity 74.4%) [11]. 

It is well known that the risk of ischemic stroke in AF patients with a low CHA_2_DS_2_VASc score is lower, and starting an oral anticoagulant in this group is not indicated [2]. Hence, looking for high-risk LAA features for thrombus to initiate oral anticoagulants among patients with low CHA_2_DS_2_VASc scores should be included in the management of stroke prevention in atrial fibrillation.

Traditionally, LAA thrombus was treated with oral VKA. The current guidelines recommend VKA with a target international normalized ratio (INR) of 2-3 in a patient with LAA thrombus, supported mainly by observational data [13,14]. However, the higher risk of bleeding among patients taking oral VKA is the main problem. As DOAC has emerged as a better oral anticoagulant for reducing bleeding risk, its use has been extended in managing LAA thrombosis. A meta-analysis comparing oral VKA and DOAC showed DOAC showed a superior likelihood of LAA thrombosis resolution [5]. In our patient, DOAC was being used to manage his LAA thrombus. Meta-analysis showed a higher chance of LAA thrombosis resolution with a lower risk of bleeding than oral VKA [5].

Catheter ablation in atrial fibrillation has been shown to have better results compared to medical therapy, especially in paroxysmal atrial fibrillation [15]. AF-related cardiomyopathy occurs when there is a vicious cycle between AF, causing atrial stunning and increased LA pressure from LV dysfunction and further adverse effects on the LA size. A study by Siow et al. showed that catheter ablation provides effective long-term control of AF-related cardiomyopathy up to a mean follow-up of 7.7 years [15]. In the patient who underwent catheter ablation for atrial fibrillation, the monitoring strategy for follow-up for AF recurrence is mixed. A recent systemic review by Unni et al. showed continuous rhythm monitoring yields a higher recurrence rate than intermittent rhythm monitoring in patients with paroxysmal AF [16]. A survey done by Schwab et al. showed that 51% of cardiologists across Europe preferred a long-term monitoring strategy and mostly recommended using self-screening, which is more cost-effective [17].

## Conclusions

A stroke due to a cardioembolic event is a devastating complication of atrial fibrillation. Oral anticoagulant is the mainstay of treatment to prevent this complication however it is only indicated for patients with high risk based on CHA_2_DS_2_VASc score. In patients with low risk based on CHA_2_DS_2_VASc score, a more detailed assessment should be performed to look for the high-risk LAA feature for thrombosis which is not included in the CHA_2_DS_2_VASc score parameters, especially in preparation for rhythm control management either cardioversion or ablation. By using transesophageal echocardiography, these high-risk LAA features can be identified and treated with oral anticoagulants.
